# Correlation between ultrawide-field fluorescence contrast results and white blood cell indexes in diabetic retinopathy

**DOI:** 10.1186/s12886-022-02442-7

**Published:** 2022-05-21

**Authors:** Lili Huang, Lele Li, Min Wang, Dongmei Zhang, Yu Song

**Affiliations:** 1 Department of Ophthalmology, Affiliated Hospital 2 of Nantong University, Nantong, 226001 People’s Republic of China; 2 Medical Research Center, Affiliated Hospital 2 of Nantong University, Nantong, 226001 People’s Republic of China

**Keywords:** Diabetes mellitus, Diabetic retinopathy, Fundus fluorescein angiography, Inflammation, Neutrophil-to-lymphocyte ratio

## Abstract

**Background:**

Diabetic retinopathy (DR) is one of the most common microvascular complications of diabetes. DR involves a state of systemic inflammation, and chronic inflammation can promote microvascular and macrovascular diseases in diabetic patients and accelerate disease progression. Ultrawide-field FFA (UWFA) systems are increasingly being used to examine a wider retina. The aim of this study was to explore the correlation between the different manifestations of retinopathy under UWFA and the systemic indicators of white blood cells in patients with diabetic retinopathy.

**Methods:**

This retrospective study included the hospitalized DR patients in the Department of Ophthalmology and Endocrinology of the Affiliated Hospital 2 of Nantong University between January 2016 and March 2019. This study examined the correlations between the UWFA examination results and glycated hemoglobin (HbA1c), routine blood tests,and the neutrophil-to-lymphocyte ratio of patients with clinically diagnosed DR during hospitalization.

**Results:**

A total of 115 patients with DR (53 females and 62 males) were included (199 eyes: 102 right eyes and 97 left eyes). UWFA revealed that most eyes (77.4%) had grade 4 microvascular leakage, 52.8% had grade 0 capillary non-perfusion area, 59.3% had grade 0 neovascularization, and 92.0% had grade 0 fibrous proliferative membranes. Microvascular leakage was correlated with the NLR (r = 0.186, *P* = 0.027). Capillary non-perfusion area was correlated with the monocyte ratio (*r* = 0.144, *P* = 0.042) and the eosinophil ratio (*r* = 0.123, *P* = 0.044). Neovascularization was correlated to the monocyte ratio (*r* = 0.324, *P* = 0.018). Finally, the fibrous proliferative membrane was correlated to the monocyte ratio (*r* = 0.418, *P* = 0.002). Only the eosinophil ratio was independently associated with proliferative DR (odds ratio = 1.25, 95% confidence interval: 1.04–1.51, *P* = 0.018).

**Conclusion:**

The results of UWFA imaging in patients with DR are correlated with white blood cell population indexes. The eosinophil ratio was independently associated with proliferative DR.

## Introduction

Chronic hyperglycemia (and other factors) can trigger biochemical and physiological changes that result in microvascular damage and retinal dysfunction [1, 2]. Diabetic retinopathy (DR) is a complication that causes blindness in diabetic patients [1, 2]. DR is the main cause of blindness in adults [1, 2]. The worldwide prevalence of DR in adults with diabetes is 34.6% [3]. The risk factors include long-duration diabetes, chronic hyperglycemia, nephropathy, hypertension, and dyslipidemia [1]. The course of the disease is difficult to reverse, and timely clinical intervention is needed [1, 2].

According to the fundus characteristics and different pathological changes, DR can be classified based on disease severity [1, 2]. Non-proliferative DR (NPDR) is characterized by retinal vascular abnormalities. Mild cases only involve microaneurysms. Moderate and severe cases involve additional vascular abnormalities. Proliferative DR (PDR) is characterized by retinal neovascularization in addition to vascular abnormalities. Diabetic macular edema is characterized by thickening of the retina near the macula.

The main tool used to distinguish between different grades of DR is fundus fluorescein angiography (FFA) [1, 2, 4]. It is a valuable diagnostic examination method. The retinal microvascular function can be observed dynamically. The abnormal proliferation of new blood vessels and fibrous tissue can be displayed, which is used to evaluate the severity of retinopathy [1, 2, 4]. In the past, traditional angiography could only show the posterior polar retina at approximately 50°. Ultrawide-field FFA (UWFA) systems are increasingly being used to examine the peripheral retina [5]. A wider range of nonperfusion areas, microvascular abnormalities, and neovascularization have been found in the peripheral retina and are involved in most DR lesions [5, 6]. In the UWFA examination system, the image features of DR patients have been regraded and analyzed to obtain a more comprehensive understanding of DR progression [5–7].

At the same time, DR involves a state of systemic inflammation, and chronic inflammation can promote microvascular and macrovascular diseases in diabetic patients and accelerate disease progression [8]. There are five kinds of white blood cells in the peripheral blood: neutrophils, eosinophils, basophils, lymphocytes, and monocytes. The neutrophils and neutrophil-to-lymphocyte ratio (NLR) can, to a certain extent, reflect the dynamic balance between inflammation and immune reactions [9]. Previous studies have shown that neutrophils, the NLR, and DR have a certain relationship with features of DR [10, 11]. Still, no studies are available for UWFA results.

Therefore, this study aimed to explore the correlation between the different manifestations of retinopathy under UWFA and the systemic indicators of white blood cells. The results could help refine the relation between retinal changes and systemic inflammation markers.

## Methods

### Study design and patients

This study complied with the Helsinki Declaration and was approved by the Ethics Committee of the Affiliated Hospital 2 of Nantong University (2021KT004). The requirement for informed consent was waived by the Ethics Committee of the Affiliated Hospital 2 of Nantong University because of the retrospective nature of the study.

This retrospective study included the hospitalized patients in the Department of Ophthalmology and Endocrinology of the Affiliated Hospital 2 of Nantong University between January 2016 and March 2019. The inclusion criteria were 1) patients clinically diagnosed with type 2 diabetes [12], 2) patients with complete hematological indicators (HbA1c, routine blood tests, blood coagulation function, liver and kidney function, etc.) during hospitalization, 3) patients who completed UWFA examination during hospitalization and were diagnosed with DR, and 4) no history of eye surgery or laser treatment. The exclusion criteria were 1) opacity of the refractive media (keratopathy, cataract, vitreous hemorrhage, etc.) that did not allow the effective observation of the angiographic image results, thus affecting the image classification and grading, 2) history of eye treatment, including retinal laser photocoagulation, intravitreal injection, vitrectomy, etc., 3) patients who underwent internal eye surgery for other non-DR diseases (such as anti-glaucoma, cataract extraction, retinal detachment surgery, etc.), 4) previous and new cases of uveitis, 5) patients with retinopathy caused by obvious hypertension, such as arteriosclerosis, arteriovenous cross indentation, etc., 6) patients with other forms of retinopathy revealed by angiography, or 7) patients with infectious or inflammatory lesions of other organs of the body and obvious abnormal routine blood tests caused by drugs, 8) patients with neurological, cardiovascular and urinary complications. Meanwhile, we retrospectively collected 80 diabetic patients without DR in the Department of Endocrinology during the same period.

### UWFA

In order to exclude contraindications, all subjects were injected with 3 ml of 20% sodium fluorescein through unilateral elbow vein, and fundus images were taken using a Heidelberg Spectralis ultrawide-angle lens (102°). The venous UWFA images of all eyes were read and reanalyzed by two physicians with more than 10 years of experience in fundus angiography diagnosis. The results of the analyses by the two doctors were compared, and the images with different judgments were re-assessed to obtain the final result.

**Fig. 1:**
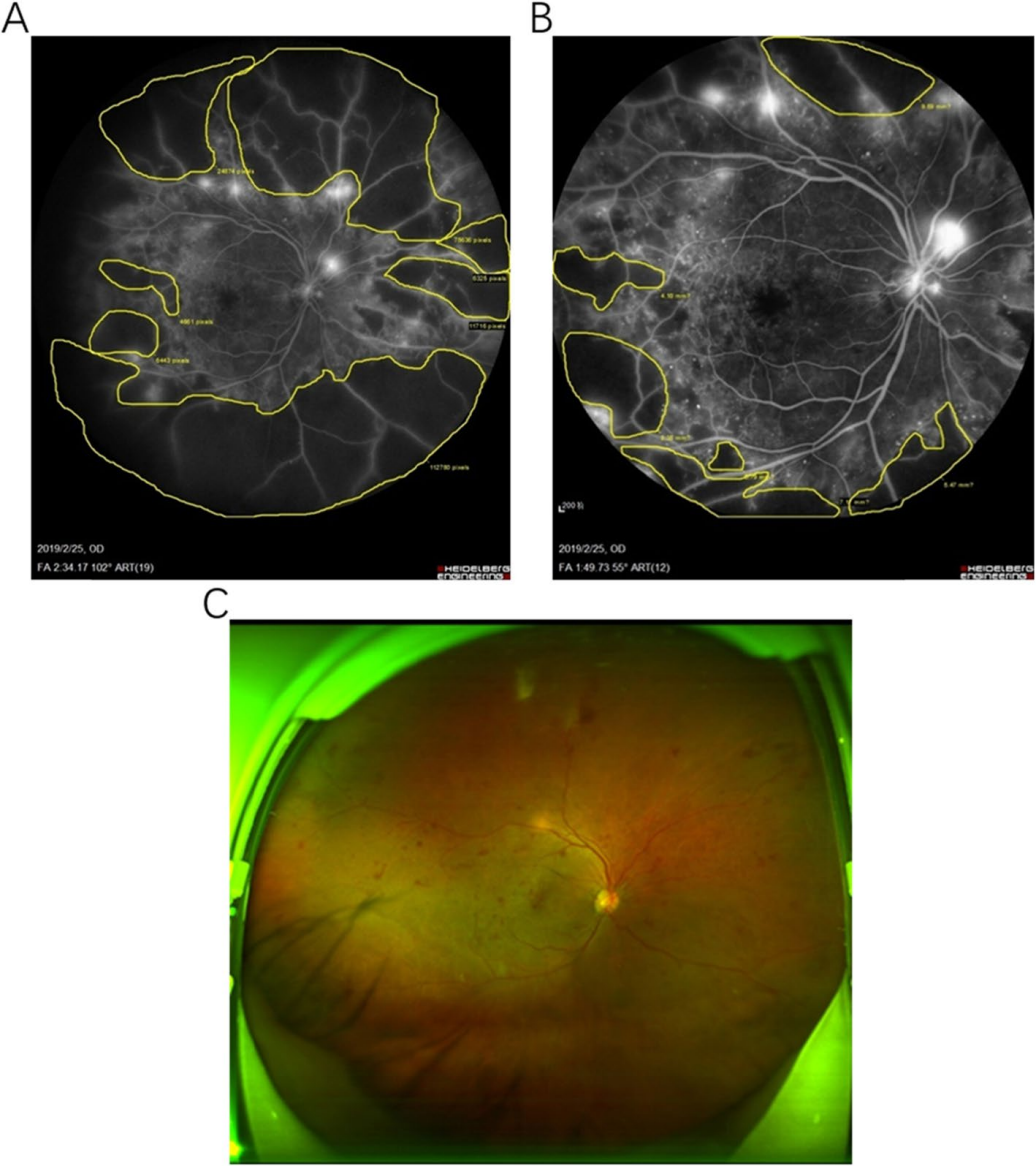
Female, 52 years old, visited the hospital for 1 week of binocular vision loss (February 22, 2019), 10 years of diabetes history, insulin control, fasting glucose 14.4 mmol/ L. **A** Fundus fluorescein angiography (FFA) of the venous phase with Heidelberg Spectralis ultrawide angle lens (102°). Dye leakage was found in microvessels in all quadrants, which was recorded as grade 4. The yellow line of the contrast instrument is the nonperfusion area of the retina, which is significantly larger than 7 DD, and it was recorded as grade 1. The optic disc and the retinal surface of each quadrant showed strong fluorescence, indicating neovascularization, recorded as grade 4. No fibrous proliferative membrane was found, grade 0. **B** FFA of the venous phase with Heidelberg 55° lens. The peripheral retina was limited. **C** Fundus photography showing the presence and exudation of the retinal surface

During the venous phase of UWFA, four image features were extracted, graded, and recorded. (1) Microvascular dye leakage. Those with no leakage anywhere in the retina were classified as grade 0, and the rest were classified as grade 1–4 according to the number of quadrants with dye leakage. (2) Capillary nonperfusion area. The capillary nonperfusion area was divided into two grades. The image measurement tools of the Heidelberg fundus camera were used. Those with a nonperfusion area less than 7 DD were rated as grade 0, and those with a nonperfusion area greater than 7 DD were rated as grade 1. (3) Neovascularization. No neovascularization of the retina or optic disc was recorded as grade 0. According to the number of quadrants in which new blood vessels were present, grades 1–4 were assigned. For optic disc neovascularization, the center of the optic disc was taken as the point of origin, and the optic disc was divided into four quadrants horizontally/vertically. The neovascularization in each quadrant of the optic disc was combined with the corresponding retinal quadrant for calculation. (4) Fiber proliferative membrane. A retina or optic disc without fibrous proliferative membrane was rated as grade 0, and those with fibrous proliferative membrane were rated as grade 1 (Fig. 1).

DR was classified as NPDR and PDR according to the International Clinical Classification of DR (2002) [13]. The core difference between PDR and NPDR is the formation of retinal neovascularization, in which the new blood vessels in the retina break through the inner boundary membrane.

### Data collection

One week before angiography, patient characteristics, glycated hemoglobin (HbA1c) levels, neutrophil values, lymphocyte values, monocyte values, and the NLR were collected.

### Statistical analysis

SPSS 23.0 (IBM, Armonk, NY, USA) was used for statistical analysis at the eye level. Spearman correlation analysis was conducted between peripheral blood indexes and UWFA image features. Logistics regression analysis was used to analyze the factors influencing PDR. Mann-Whitney U test was used to analyze defferences between the diabetic patients without DR group and DR group. Variables with *P* < 0.10 in the univariable analyses were included in the multivariable analysis (enter method). *P*-values < 0.05 were considered statistically significant.

## Results

### Characteristics of the patients and eyes

The study included 115 patients (53 females and 62 males). There were 199 eyes; 102 right eyes and 97 left eyes were examined. The mean age was 56.7 ± 8.9 years. The course of diabetes was 10 years. Fifty-four patients had a history of hypertension. There were 118 cases of NPDR in 199 eyes, including 15 mild NPDR eyes, 10 moderate NPDR eyes, and 93 severe NPDR eyes. PDR was present in 81 eyes (Table 1).

**Table 1:** Characteristics of the patients

Characteristics	*n* = 115
Age (years)	56.7 ± 8.9
Sex (male/female)	62/53
Course of diabetes (years)	10 (1, 30)
HbA1c (%)	9.6 (5.9, 15.4)
Hypertension, n (%)	54 (47.0)
Course of hypertension (years)	2.0 (0, 40)
Number of eyes, n (%)	199
NPDR	118 (59.3)
Mild	15 (12.7)
Moderate	10 (8.5)
Severe	93 (78.8)
PDR	81 (40.7)
White blood cell count (× 10^9^/L)	6.50 (3.30, 10.30)
Neutrophil ratio (%)	60.90 (5.00, 83.00)
Lymphocyte ratio (%)	29.10 (9.30, 48.90)
Monocyte ratio (%)	6.40 (1.40, 11.90)
Basophil ratio (%)	0.40 (0.00, 5.70)
Eosinophil ratio (%)	1.60 (0.10, 8.80)
NLR (%)	2.14 (0.78, 40.41)

*HbA1c* Glycated hemoglobin, *NPDR* Non-proliferative diabetic retinopathy, *PDR* Proliferative diabetic retinopathy, *NLR* Neutrophil-to-lymphocyte ratio

### UWFA examination

UWFA revealed that most eyes (77.4%) had grade 4 microvascular leakage, 52.8% had grade 0 capillary non-perfusion area, 59.3% had grade 0 neovascularization, and 92.0% had grade 0 fibrous proliferative membranes (Table 2).

**Table 2:** Characteristic classification of UWFA in diabetic retinopathy

Image features	Grade 0	Grade 1	Grade 2	Grade 3	Grade 4
Microvascular leakage	19 (9.5)	13 (6.5)	4 (2.0)	9 (4.5)	154 (77.4)
Capillary nonperfusion area	105 (52.8)	94 (47.2)	–	–	–
Neovascularization	118 (59.3)	32 (16.1)	19 (9.5)	14 (7.0)	16 (8.0)
Fibrous proliferative membrane	183 (92.0)	16 (8.0)	–	–	–

Data are presented as n (%)

### Correlation analysis

Microvascular leakage was correlated with the NLR (*r* = 0.186, *P* = 0.027). Capillary non-perfusion area was correlated with the monocyte ratio (*r* = 0.144, *P* = 0.042) and the eosinophil ratio (*r* = 0.123, *P* = 0.044). Neovascularization was correlated to the monocyte ratio (*r* = 0.324, *P* = 0.018). Finally, fibrous proliferative membrane was correlated to the monocyte ratio (*r* = 0.418, *P* = 0.002) (Table 3).

**Table 3:** Correlation between peripheral blood indexes and wide-angle FFA image features

	White blood cell count (r, P)	Neutrophil ratio (r, P)	Lymphocyte ratio (r, P)	Monocyte ratio (r, P)	Basophil ratio (r, P)	Eosinophil ratio (r, P)	NLR (r, P)
Microvascular leakage	0.049, 0.488	0.150, 0.048	0.051, 0.476	0.105, 0.039	0.002, 0.983	−0.036, 0.610	0.186, 0.027
Capillary nonperfusion area	0.067, 0.346	0.029, 0.686	0.023, 0.747	0.144, 0.042	−0.092, 0.196	0.123, 0.044	−0.028, 0.693
Neovascularization	0.048, 0.500	0.068, 0.339	−0.024, 0.732	0.324, 0.018	−0.079, 0.269	0.097, 0.174	0.023, 0.742
Fibrous proliferative membrane	0.005, 0.946	0.027, 0.701	0.009, 0.900	0.418, 0.002	−0.055, 0.440	−0.007, 0.923	− 0.045, 0.529

*NLR* Neutrophil-to-lymphocyte ratio

### Difference analysis

The patients were divided into two groups: 80 diabetic patients without DR, and 115 patients with DR. There were differences in white blood cell count (*P* = 0.004), monocyte ratio (*P* = 0.002) and eosinophils ratio (*P* = 0.042) in diabetic patients without DR group and DR group.(Table 4).

**Table 4:** Comparison between diabetic patients without Dr. group and Dr. group

Variables	DM(80)	DR(115)	*p*-Value
Age (years)	52 ± 9.5	56.7 ± 8.9	–
Sex (male/female)	48/32	62/53	–
Course of diabetes (years)	4 (1,11)	10 (1, 30)	–
HbA1c (%)	8.2 (4.8, 13.4)	9.6 (5.9, 15.4)	–
Hypertension, n (%)	36 (45.0)	54 (47.0)	–
Course of hypertension (years)	1.6 (0,30)	2.0 (0, 40)	–
White blood cell count (×10^9^/L)	5.20 (3.80, 7.30)	6.50 (3.30, 10.30)	0.04
Neutrophil ratio (%)	57.54 (52.10,77.10)	60.90 (5.00, 83.00)	0.132
Lymphocyte ratio (%)	29.04 (10.50,46.50)	29.10 (9.30, 48.90)	0.964
Monocyte ratio (%)	5.26 (1.50, 10.80)	6.40 (1.40, 11.90)	0.002
Basophil ratio (%)	0.46 (0.00, 3.72)	0.40 (0.00, 5.70)	0.585
Eosinophil ratio (%)	1.14 (0.20, 6.60)	1.60 (0.10, 8.80)	0.042
NLR (%)	2.02 (0.80, 32.80)	2.14 (0.78, 40.41)	0.504

### Factors influencing PDR

The univariable analyses showed that sex (*P* = 0.085), the monocyte ratio (*P* = 0.032), and the eosinophil ratio (*P* = 0.040) could be entered into the multivariable analysis. Only the eosinophil ratio was independently associated with PDR (OR = 1.25, 95%CI: 1.04–1.51, *P* = 0.018) (Table 5).

**Table 5:** Influencing factors of PDR

Variable	Univariable analyses			Multivariable analysis		
	OR	95%CI	*P*	OR	95%CI	*P*
Age	0.997	0.965–1.029	0.833			
Sex	1.658	0.933–2.919	0.085	1.662	0.914–3.023	0.096
Course of diabetes	1.026	0.983–1.071	0.240			
HbA1c	0.985	0.850–1.142	0.845			
Hypertension	1.197	0.679–2.109	0.535			
Course of hypertension	1.009	0.969–1.051	0.672			
White blood cell count	1.076	0.900–1.286	0.423			
Neutrophil ratio	1.018	0.987–1.050	0.255			
Lymphocyte ratio	0.999	0.963–1.036	0.941			
Monocyte ratio	0.872	1.748–2.018	0.032	0.854	0.724–1.008	0.062
Eosinophil ratio	1.169	1.081–1.392	0.040	1.250	1.039–1.505	0.018
Basophil ratio	1.281	0.634–2.587	0.491			
NLR	0.971	0.874–1.079	0.585			

*OR* Odds ratio, *CI* Confidence interval, *HbA1c* Glycated hemoglobin, *NLR* Neutrophil-to-lymphocyte ratio

## Discussion

Previous studies have shown that neutrophils, the NLR, and DR have a certain relationship with features of DR [10, 11]. Still, no studies are available for UWFA results. Therefore, this study aimed to explore the correlation between the different manifestations of retinopathy under UWFA and the systemic indicators of white blood cells. This study retrospectively analyzed the different features of UWFA images in DR patients and observed retinal microvascular lesions more extensively and deeply, reflecting the process of disease progression. Each image feature was graded and compared with the peripheral blood leukocyte system. The results suggest that the features of UWFA imaging in patients with DR are correlated with white blood cell population indexes. The study results further confirmed the inflammatory injury mechanism of DR and provided some insight for us to predict the degree of DR microvascular disease from the level of circulating white blood cells.

Many DR lesions are related to the peripheral retina, and a broader observation of the peripheral retina is very important for the screening, diagnosis, treatment, and prognosis of the disease [14]. However, conventional angiography can only show 30°-50° of the retinal surface in a single view. For patients with DR, UWFA provides a better view of the peripheral retina than conventional fundus angiography [15]. A retrospective study showed that UWFA could display an angiographic sign associated with DR known as peripheral vascular leakage (PVL), which is a late leakage of dye in the retinal arteries and veins, reflecting the breakdown of the blood-retinal barrier (BRB) in active retinopathy. In this hospital and during the study period, a Heidelberg Spectralis ultrawide field lens (102°) [16] was used for UWFA to observe a wider range of peripheral retinal changes and obtain a broader understanding of DR. On this basis, the four main imaging features (microvascular leakage, nonperfusion, neovascularization, and proliferative membranes) were analyzed. The dye leakage of retinal arteries and veins suggested the destruction of the BRB. When the nonperfusion of the retina is greater than 7 DD, new blood vessels can be generated and are proportional to the size of the nonperfusion area [17]. Therefore, in this study, 7 DD was used as the basis for classifying nonperfusion areas in UWFA images. The progression of DR involves a long period of nonperfusion and hypoxia that induces new blood vessels and fibroproliferative membranes.

After tissue migration and activation of peripheral blood neutrophils under pathological conditions, cytokines, chemokines, matrix metalloproteinases, and other substances can be secreted, leading to tissue damage and immune cell infiltration [18]. Some phenotypes also inhibit lymphocytes [19], which can promote the occurrence of chronic inflammation. To a certain extent, the NLR can reflect the dynamic balance between inflammation and the immune response [9, 11]. Previous studies have suggested that neutrophils impair the integrity of the retinal pigment epithelial barrier [20]. In this study, microvascular dye leakage in UWFA images suggested BRB destruction and vascular endothelial function damage. The vascular endothelium performs important functions, including managing microvascular permeability, coagulation, inflammation, vascular tension, and neovascularization [21]. Diabetes mellitus, hypercholesterolemia, and hypertension lead to a dysregulation of vascular endothelial L-arginine/nitric oxide synthase (eNOS) and resulting in vascular endothelial dysfunction [22]. HbA1c is strongly associated with increased levels of circulating adhesion molecules (ICAM, VCAM, etc.), which are considered to be indicators of endothelial cell injury and are correlated with the severity of DR [23]. This study showed that microvascular dye leakage was correlated with the proportion of neutrophils, monocytes, and the NLR in peripheral blood leukocytes. The results were therefore consistent with previous studies.

Damaged capillary endothelial cells, reduced tissue perfusion and oxygenation, and aggravated vascular damage occlusion resulted in no perfusion on UWFA. Schroder et al. [24] proposed for the first time in 1991 the concept that activated white blood cells lead to capillary obstruction and proved that granulocytes and monocytes were trapped in retinal capillaries. Compared with normal cells, white blood cells in diabetic patients, especially those in capillaries, release more reactive oxygen species, damage endothelial cells, and pericytes, and lead to retinopathy through oxidative stress [25, 26]. Elevated levels of inflammatory mediators caused by the accumulation of advanced glycation end products can also lead to persistent chronic inflammation of the retina, resulting in the activation of white blood cells, adherence to vascular endothelium, and extravasation [27, 28]. In addition, there is evidence that elevated white blood cell count within the normal range is associated with the occurrence of microvascular and macrovascular complications of diabetes [29]. The results showed that the degree of capillary occlusion was correlated with the proportion of monocytes and eosinophils in peripheral blood. These conditions indicate that the retina progresses from vascular endothelial cell damage in the early stage to capillary occlusion in the later stage, and the ratio of eosinophils and monocytes in peripheral blood might reflect the course of the disease.

Monocytes are one of the major leukocyte subtypes and are also considered markers of inflammation [30]. Animal studies have shown that when many monocytes enter the retinal tissue, the retinal pigment epithelium acts as a channel for monocytes to transport to the retina [31, 32]. Studies have shown that plasma levels of monocyte chemotactic protein-1 (MCP-1), which regulates monocyte chemotactic and inflammatory processes, are significantly increased in patients with DR [33]. There is also an association between peripheral blood monocyte levels and the prevalence of DR [34]. In this study, the range of retinal neovascularization and the presence of fibroproliferative membrane in UWFA images were significantly correlated with the proportion of peripheral blood mononuclear cells. Studies have shown that monocytes and macrophages might be involved in the process of angiogenesis in atherosclerosis [35, 36]. Monocytes in circulating blood are considered precursors to tissue macrophages, whose function is to transform into a variety of tissue-sensing macrophages during normal homeostasis and inflammation. After tissue injury, inflammation and macrophage aggregation are induced, and macrophage proliferation in situ and synthesis of vascular endothelial growth factors (VEGFs) are promoted. Subsequently, it promotes the growth of new blood vessels and activates fibroblasts to produce collagen [37]. During DR progression, chronic inflammation leads to microvascular injury, which goes through the damage repair process involving macrophages. This process explains the results of this study well. The ratio of monocytes in peripheral blood is correlated with the degree of retinal neovascularization and proliferative fibrous membrane.

PDR is the final stage in the progression of DR and will lead to blindness [1, 2]. In the present study, only the eosinophil ratio was independently associated with PDR. Diabetes involves a dysregulated immunity, including eosinophils [38]. Elevated eosinophil counts have been observed in both men and women with PDR [39]. PDR might involve eosinophil-specific chemokines that play important roles in the inflammation observed in DR [40].

This study has some limitations. The sample size was small, and the patients were from a single center, leading to the possibility of regional differences. In this study, leukocyte-related indicators were studied, but other inflammatory markers in peripheral blood were not studied. In addition, the white blood cells could not be used to evaluate the damage of other diabetic target organs completely. The traditional direct evaluation of intraocular fluid is more valuable, but detecting inflammatory factors in intraocular fluid such as aqueous humor was not done for comparison. The extraction of intraocular fluid for detection is invasive, and a more safe, economical, and valuable detection method is necessary.

## Conclusion

In the UWFA of DR, microvascular leakage was correlated with the NLR, the capillary non-perfusion area was correlated with the monocyte ratio and the eosinophil ratio, neovascularization was correlated to the monocyte ratio, and the fibrous proliferative membrane was correlated to the monocyte ratio. Therefore, the neutrophil ratio, monocyte ratio, eosinophil ratio, and NLR could be are of great value for in-depth understanding of the evolution of retinal microvascular disease in DR patients.. In addition, the eosinophil ratio was associated with PDR. Further studies are necessary to determine their predictive and diagnostic values.

## Data Availability

All data are available under request. Yu Song should be contacted if someone wants to request the data.

## Abbreviations

DR: Diabetic retinopathy
FFA: Fluorescein angiography
UWFA: Ultrawide-field FFA
NPDR: Non-proliferative DR
PDR: Proliferative DR
NLR: Neutrophil-to-lymphocyte ratio

## References

[1] Wong TY, Cheung CM, Larsen M (2016). Diabetic retinopathy. Nat Rev Dis Primers.

[2] Wong TY, Sun J, Kawasaki R (2018). Guidelines on diabetic eye care: the International Council of Ophthalmology Recommendations for screening, follow-up, referral, and treatment based on resource settings. Ophthalmology..

[3] Yau JW, Rogers SL, Kawasaki R (2012). Global prevalence and major risk factors of diabetic retinopathy. Diabetes Care.

[4] Tran K, Pakzad-Vaezi K (2018). Multimodal imaging of diabetic retinopathy. Curr Opin Ophthalmol.

[5] Kumar V, Surve A, Kumawat D (2021). Ultra-wide field retinal imaging: a wider clinical perspective. Indian J Ophthalmol.

[6] Jones NP, Sala-Puigdollers A, Stanga PE (2017). Ultra-widefield fundus fluorescein angiography in the diagnosis and management of retinal vasculitis. Eye (Lond).

[7] Rabiolo A, Parravano M, Querques L (2017). Ultra-wide-field fluorescein angiography in diabetic retinopathy: a narrative review. Clin Ophthalmol.

[8] Fujita T, Hemmi S, Kajiwara M (2013). Complement-mediated chronic inflammation is associated with diabetic microvascular complication. Diabetes Metab Res Rev.

[9] Faria SS, Fernandes PC, Silva MJ (2016). The neutrophil-to-lymphocyte ratio: a narrative review. Ecancer Med Sci.

[10] Woo SJ, Ahn SJ, Ahn J (2011). Elevated systemic neutrophil count in diabetic retinopathy and diabetes: a hospital-based cross-sectional study of 30,793 Korean subjects. Invest Ophthalmol Vis Sci.

[11] Liu J, Liu X, Li Y (2018). The association of neutrophil to lymphocyte ratio, mean platelet volume, and platelet distribution width with diabetic retinopathy and nephropathy: a meta-analysis. Biosci Rep.

[12] Chatterjee S, Khunti K (2017). Davies MJ type 2 diabetes. Lancet.

[13] Wilkinson CP, Ferris FL, Klein RE (2003). Proposed international clinical diabetic retinopathy and diabetic macular edema disease severity scales. Ophthalmology..

[14] Soliman AZ, Silva PS, Aiello LP (2012). Ultra-wide field retinal imaging in detection, classification, and management of diabetic retinopathy. Semin Ophthalmol.

[15] Oliver SC, Schwartz SD (2010). Peripheral vessel leakage (PVL): a new angiographic finding in diabetic retinopathy identified with ultra wide-field fluorescein angiography. Semin Ophthalmol.

[16] Witmer MT, Parlitsis G, Patel S (2013). Comparison of ultra-widefield fluorescein angiography with the Heidelberg Spectralis((R)) noncontact ultra-widefield module versus the Optos((R)) Optomap((R)). Clin Ophthalmol.

[17] Zhang H (2012). Laser treatment for ocular fundus diseases.

[18] Kruger P, Saffarzadeh M, Weber AN (2015). Neutrophils: between host defence, immune modulation, and tissue injury. PLoS Pathog.

[19] Pillay J, Kamp VM, van Hoffen E (2012). A subset of neutrophils in human systemic inflammation inhibits T cell responses through mac-1. J Clin Invest.

[20] Zhou J, He S, Zhang N (2010). Neutrophils compromise retinal pigment epithelial barrier integrity. J Biomed Biotechnol.

[21] Endemann DH, Schiffrin EL (2004). Endothelial dysfunction. J Am Soc Nephrol.

[22] Jamwal S, Sharma S (2018). Vascular endothelium dysfunction: a conservative target in metabolic disorders. Inflamm Res.

[23] Tryggestad JB, Shah RD, Braffett BH (2020). Circulating adhesion molecules and associations with HbA1c, hypertension, nephropathy, and retinopathy in the treatment options for type 2 diabetes in adolescent and youth study. Pediatr Diabetes.

[24] Schroder S, Palinski W, Schmid-Schonbein GW (1991). Activated monocytes and granulocytes, capillary nonperfusion, and neovascularization in diabetic retinopathy. Am J Pathol.

[25] Griendling KK, Sorescu D, Ushio-Fukai M (2000). NAD(P) H oxidase: role in cardiovascular biology and disease. Circ Res.

[26] Scott JA, King GL (2004). Oxidative stress and antioxidant treatment in diabetes. Ann N Y Acad Sci.

[27] Kim SY, Johnson MA, McLeod DS (2005). Neutrophils are associated with capillary closure in spontaneously diabetic monkey retinas. Diabetes..

[28] Joussen AM, Murata T, Tsujikawa A (2001). Leukocyte-mediated endothelial cell injury and death in the diabetic retina. Am J Pathol.

[29] Tong PC, Lee KF, So WY (2004). White blood cell count is associated with macro- and microvascular complications in chinese patients with type 2 diabetes. Diabetes Care.

[30] Badr RE, Salama MI, Abd-Elmaogood AK (2019). Toll-like receptor 2 expression on monocytes and microvascular complications in type 2 diabetic patients. Diabetes Metab Syndr.

[31] Benhar I, Reemst K, Kalchenko V (2016). The retinal pigment epithelium as a gateway for monocyte trafficking into the eye. EMBO J.

[32] Rangasamy S, McGuire PG, Franco Nitta C (2014). Chemokine mediated monocyte trafficking into the retina: role of inflammation in alteration of the blood-retinal barrier in diabetic retinopathy. PLoS One.

[33] Jiang Z, Hennein L, Xu Y (2016). Elevated serum monocyte chemoattractant protein-1 levels and its genetic polymorphism is associated with diabetic retinopathy in Chinese patients with type 2 diabetes. Diabet Med.

[34] Wan H, Cai Y, Wang Y (2020). The unique association between the level of peripheral blood monocytes and the prevalence of diabetic retinopathy: a cross-sectional study. J Transl Med.

[35] Jaipersad AS, Lip GY, Silverman S (2014). The role of monocytes in angiogenesis and atherosclerosis. J Am Coll Cardiol.

[36] Moroni F, Ammirati E, Norata GD (2019). The role of monocytes and macrophages in human atherosclerosis. Plaque Neoangiogenesis, and Atherothrombosis. Mediat Inflamm.

[37] Kotwal GJ, Chien S (2017). Macrophage differentiation in Normal and accelerated wound healing. Results Probl Cell Differ.

[38] Xu H, Chen M (2017). Diabetic retinopathy and dysregulated innate immunity. Vis Res.

[39] Fukui M, Tanaka M, Hamaguchi M (2009). Eosinophil count is positively correlated with albumin excretion rate in men with type 2 diabetes. Clin J Am Soc Nephrol.

[40] Schoenberger SD, Kim SJ, Sheng J (2012). Increased prostaglandin E2 (PGE2) levels in proliferative diabetic retinopathy, and correlation with VEGF and inflammatory cytokines. Invest Ophthalmol Vis Sci.

